# Unique photosynthetic phenotypes in *Portulaca* (Portulacaceae): C_3_-C_4_ intermediates and NAD-ME C_4_ species with Pilosoid-type Kranz anatomy

**DOI:** 10.1093/jxb/erw393

**Published:** 2016-12-15

**Authors:** Elena V Voznesenskaya, Nuria K Koteyeva, Gerald E Edwards, Gilberto Ocampo

**Affiliations:** 1 Laboratory of Anatomy and Morphology, VL Komarov Botanical Institute of Russian Academy of Sciences, St. Petersburg, Russia; 2 School of Biological Sciences, Washington State University, Pullman, WA, USA; 3 Universidad Autónoma de Aguascalientes, Aguascalientes, Mexico

**Keywords:** C_3_ plants, C_3_-C_4_ intermediate, C_4_ plants, chloroplast ultrastructure, immunolocalization, NAD-ME type, NADP-ME type, photosynthetic enzymes, *Portulaca*, Portulacaceae

## Abstract

Portulacaceae is a family that has considerable diversity in photosynthetic phenotypes. It is one of 19 families of terrestrial plants where species having C_4_ photosynthesis have been found. Most species in *Portulaca* are in the alternate-leaved (AL) lineage, which includes one clade (Cryptopetala) with taxa lacking C_4_ photosynthesis and three clades having C_4_ species (Oleracea, Umbraticola and Pilosa). All three species in the Cryptopetala clade lack Kranz anatomy, the leaves have C_3_-like carbon isotope composition and they have low levels of C_4_ cycle enzymes. Anatomical, biochemical and physiological analyses show they are all C_3_-C_4_ intermediates. They have intermediate CO_2_ compensation points, enrichment of organelles in the centripetal position in bundle sheath (BS) cells, with selective localization of glycine decarboxylase in BS mitochondria. In the three C_4_ clades there are differences in Kranz anatomy types and form of malic enzyme (ME) reported to function in C_4_ (NAD-ME versus NADP-ME): Oleracea (Atriplicoid, NAD-ME), Umbraticola (Atriplicoid, NADP-ME) and Pilosa (Pilosoid, NADP-ME). Structural and biochemical analyses were performed on Pilosa clade representatives having Pilosoid-type leaf anatomy with Kranz tissue enclosing individual peripheral vascular bundles and water storage in the center of the leaf. In this clade, all species except *P. elatior* are NADP-ME-type C_4_ species with grana-deficient BS chloroplasts and grana-enriched M chloroplasts. Surprisingly, *P. elatior* has BS chloroplasts enriched in grana and NAD-ME-type photosynthesis. The results suggest photosynthetic phenotypes were probably derived from an ancestor with NADP-ME-type C_4_, with two independent switches to NAD-ME type.

## Teaser

Portulacaceae shows great diversity in C_4_ photosynthetic phenotypes: all species in clade Cryptopetala are C_3_-C_4_ intermediates, while clade Pilosa has a unique anatomical form of Kranz with diversity in C_4_ biochemistry.

## Introduction

Most terrestrial plants have C_3_-type photosynthesis, in which there is direct fixation of atmospheric CO_2_ via ribulose bisphosphate carboxylase oxygenase (Rubisco) in all chloroplast-containing leaf mesophyll (M) tissues. Only ~3% of terrestrial plant species are C_4_, in which atmospheric CO_2_ is fixed by phosphoenolpyruvate carboxylase (PEPC) located in M cells, with the production of initial compounds containing four carbon atoms. Through the CO_2_ concentrating mechanism and repression of photorespiration, C_4_ plants are recognized as having an increased capacity for carbon assimilation and higher efficiency in nitrogen and water use in warm climates. For this reason, there is interest in genetically modifying major C_3_ crops with C_4_ traits to reduce photorespiration (Brown, 1999; Sheehy *et al.*, 2007). The main structural difference associated with this pathway in most C_4_ species is the specialized leaf anatomy (called Kranz type) with close coordination of function between two types of cells surrounding vascular bundles (VB), the enlarged chlorenchymatous bundle sheath (BS) cells and the radially arranged M cells (Edwards and Walker, 1983; Hatch, 1987; Kanai and Edwards, 1999, Edwards and Voznesenskaya, 2011). C_4_ photosynthesis has been found in 19 families of angiosperm plants, 16 of which correspond to dicot lineages (Sage, 2004).

In the order Caryophyllales there are 23 families (Thorne and Reveal, 2007), eight of which have species with C_4_ photosynthesis: Aizoaceae, Amaranthaceae, Caryophyllaceae, Gisekiaceae, Molluginaceae, Nyctaginaceae, Polygonaceae and Portulacaceae (Sage, 2004). Portulacaceae has 29 genera as traditionally circumscribed (Eggli, 2002), although it is currently considered as a family with only one genus, *Portulaca* (Nyffeler and Eggli, 2010; Hernández-Ledesma *et al.*, 2015), which is known to have C_4_ species. Previous suggestions that some species of *Anacampseros* and *Grahamia* (once considered as members of Portulacaceae but now circumscribed in Anacampserotaceae) may be C_4_ are not supported by a recent study (Guralnick *et al.*, 2008), which indicates the occurrence of Crassulacean acid metabolism (CAM) rather than C_4_ photosynthesis in these genera.

For many years it was accepted that the genus *Portulaca* includes only species having Kranz-type anatomy and C_4_ photosynthesis with two clear groups. One group was defined as having species with NAD-malic enzyme (NAD-ME)-type C_4_ cycle and well-developed grana in BS cell chloroplasts, as in *P. oleracea* L. (Laetsch, 1971, 1974; Gutierrez *et al.*, 1974; Carolin *et al.*, 1978; Sprey and Laetsch, 1978). Another group was described as having NADP-malic enzyme (NADP-ME) subtype species with agranal BS chloroplasts, with *P. grandiflora* Hook. being a well-known representative species (Gutierrez *et al.*, 1974; Carolin *et al.*, 1978). This diversity was a stimulus for more complex studies of representative species in the genus. A structural and functional analysis showed the existence of four clearly distinct C_4_ types of leaf anatomy, which differ in the way Kranz anatomy is formed with respect to the position of the VB (Voznesenskaya *et al.*, 2010). Phylogenetic analyses of the suborder Cactineae, which included genus *Portulaca*, showed the existence of two clearly defined clades; the opposite-leaved clade (OL), and the alternate-leaved clade (AL), mostly corresponding to two previously recognized subgenera, *Portulacella* and *Portulaca* (Ocampo and Columbus, 2010, 2012). There are two well-defined clades within the OL clade representing Australian and African-Asian species (Ocampo and Columbus, 2012). All representatives which have been studied in the OL clade have NADP-ME-type biochemistry, and a unique form of leaf anatomy (Portulacelloid type) where Kranz is formed around individual VB, which are located towards the adaxial side of the leaf, with several layers of water storage (WS) cells located towards the abaxial side (Voznesenskaya *et al.*, 2010; Ocampo *et al.*, 2013). In the AL clade, from studies of representative species, there are four clades with differences in anatomy and forms of photosynthesis: Oleracea, Umbraticola, Pilosa and Cryptopetala. The Umbraticola clade is reported to have NADP-ME species with Atriplicoid-type anatomy where Kranz forms around individual veins in planar leaves. The Oleracea clade has NAD-ME-type C_4_ species with a specific variant of Atriplicoid-type leaf anatomy (Voznesenskaya *et al.*, 2010). The Pilosa clade is reported to have NADP-ME-type C_4_ species with Pilosoid-type anatomy in which Kranz tissue encloses individual peripheral VB with WS tissue located in the center of the leaf. In the Cryptopetala clade, which is sister to the Oleracea clade, one species, *P. cryptopetala*, was shown to be a C_3_-C_4_ intermediate species (Voznesenskaya *et al.*, 2010); *P. hirsutissima* and *P. mucronata* are two other species in this clade which have C_3_-type carbon isotope composition, and from examination of leaf lamina of herbarium specimens, an apparent lack of Kranz anatomy (Ocampo *et al.*, 2013). Since evolution of C_4_ from C_3_ species is considered a stepwise process with intermediate states (Sage *et al.*, 2012), the goal of the present work was to fully characterize photosynthesis in species in clade Cryptopetala, and to analyze forms of photosynthesis in representative species in the under-studied Pilosa clade, which has a form of Kranz only found in Portulacaceae (Voznesenskaya *et al.*, 2010) and Aizoaceae (Bohley *et al.*, 2015).

## Materials and methods

### Plant material

The sources of seeds and plants of species in this study are provided in Table 1. All seeds were stored at 3–5 ºC prior to use and were germinated on the surface of potting soil (Sunshine LC-1 from SUNGRO Horticulture, Bellevue, WA, USA) at 25 ºC and a photosynthetic photon flux density (PPFD) of 100 µmol quanta m^-2^ s^-1^. The seedlings were then transplanted to soil in 10 cm diameter pots (one seedling per pot). After ~ 1 week established plants were transferred to a greenhouse with day/night temperatures 26/18 °C and a maximum mid-day PPFD of 1000 µmol photosynthetic quanta m^−2^s^−1^. Plants were fertilized once per week with Peter’s Professional (20:20:20; Scotts Miracle-Gro). For microscopy and biochemical analyses, samples of mature leaves were taken from ~2-month-old plants. Cotyledons were fixed ~2 weeks after germination when the first leaves were already established.

**Table 1.** The source of seeds and carbon isotope composition, δ^13^C, for mature leaves. For δ^13^C analysis the minimum is *n*=2

Species	Source of plants	δ^13^C,^o^/_oo_
**Aizoaceae**		
*Sesuvium portulacastrum* (L.) L.	Cuttings from Honolulu, Hawaii, USA	−29.5 ± 0.07*
**Cryptopetala clade**		
*P. cryptopetala* Speg.	Ulm Botanical Garden, Germany 2009/474 IPEN UY-)-ULM-2008-G-82 (Sukkulentenzamlung Zürich, from Uruguay, Negro Ruta, Nyffeler & Eggli), (under the name *P. fluvialis*)	−28.6 ± 0.16
*P. hirsutissima* Cambess.	Plants provided by R. Nichloson, Smith College, Northampton, MA	−26.9 ± 0.08
*P. mucronata* Link.	Seeds provided by G. Pinna-de-Melo, Universidade de São Paulo, São Paulo, Brazil	−26.6 ± 0.54
**Oleracea clade**		
*P. molokiniensis* Hobdy	Seeds provided by F. Okamoto, Pearl City, Hawaii	−15.4 ± 0.37*
*P. oleracea* L.	Seeds were collected in Pullman, WA	−14.0 ± 0.83*
**Umbraticola clade**		
*P. umbraticola* Kunth. cv. ‘wildfire mixed’	Philippines, plant market	−13.5 ± 0.29*
**Pilosa clade**		
*P. amilis* Speg.	Royal Botanic Gardens, Kew, #6541	−13.5 ± 0.03*
*P. biloba* Urb.	Seeds from J. Matthews (Carter and Snow 21355a, herbarium UAA)	−13.8 ± 0.08
*P. elatior* Mart. ex Rohrb.	Seeds provided by G. Pinna-de-Melo, Universidade de São Paulo, São Paulo, Brazil	−13.0 ± 0.08
*P.* cf. *gilliesii* Hook.	Plants provided by R. Nicholson, Smith College, Northampton, MA	−17.4 ± 0.01
*P. grandiflora* Hook.	Lilly Miller, The Chas. H. Lilly Co., Portland, OR	−12.1 ± 0.57*
*P. halimoides* L.	Seeds provided by R. Nicholson, Smith College, Northampton, MA	−16.6 ± 1.36
*P. smallii* P. Wilson	Seeds from G. Ocampo (Matthews and Luckenbaugh s.n., 30 Aug 2013, herbarium UAA)	−13.9 ± 0.25
*P. suffrutescens* Engelm.	Seeds from USDA PI674272	−13.2 ± 0.38

*Data from (Voznesenskaya *et al.*, 2010)

### Light and electron microscopy

Hand cross sections of fresh leaves were placed in water and studied under UV light (with DAPI filter) on a Leica Fluorescence Microscope Leica DMFSA (Leica Microsystems Wetzlar GmbH, Germany).

For structural studies, two to three samples were taken from three plants for each species from cotyledons and the middle part of the leaf. They were fixed at 4 ^o^C in 2% (v/v) paraformaldehyde and 2% (v/v) glutaraldehyde in 0.1 M phosphate buffer (pH 7.2), postfixed in 2% (w/v) OsO_4_ and then, after a standard acetone dehydration procedure, embedded in Spurr’s epoxy resin. Cross sections were made on a Reichert Ultracut R ultramicrotome (Reichert-Jung GmbH, Heidelberg, Germany). For light microscopy, semi-thin sections were stained with 1% (w/v) Toluidine blue O in 1% (w/v) Na_2_B_4_O_7_ and studied under the Olympus BH-2 (Olympus Optical Co., Ltd.) light microscope equipped with LM Digital Camera & Software (Jenoptik ProgRes Camera, C12plus, Jena, Germany). Ultra-thin sections were stained for transmission electron microscopy with 4% (w/v) uranyl acetate followed by 2% (w/v) lead citrate. FEI Tecnai G2 (Field Emission Instruments Company, Hillsboro, OR, USA) equipped with Eagle FP 5271/82 4K HR200KV digital camera and Hitachi H-600 (Hitachi Scientific Instruments, Tokyo, Japan) transmission electron microscopes were used for observation and photography.

Observations of vascular pattern were obtained from fully expanded leaves. The samples were cleared in 70% ethanol (v/v) until chlorophyll was removed, treated with 5% (w/v) NaOH overnight and then rinsed three times in water. At least five leaves from two or three different plants were used. The leaves were mounted in water and examined under UV light (with DAPI filter) on a Fluorescence Microscope Leica DMFSA (Leica Microsystems Wetzlar GmbH, Germany) using autofluorescence of lignified tracheary elements of the xylem. The density of the venation (mm per mm^2^ of the leaf surface area) was determined as the length of minor or peripheral veins per leaf area which were measured using the image analysis program ImageJ 1.37v (Wayne Rasband, National Institutes of Health, USA). Standard errors were determined and analysis of variance (ANOVA) was performed with Statistica 7.0 software (StatSoft, Inc.). Tukey’s HSD (honest significant difference) test was used to analyze differences between vein density values in *Portulaca* species. All analyses were performed at the 95% significance level.

### *In situ* immunolocalization

Leaf samples (two to three samples from three plants for each species) were fixed at 4 ^o^C in 2% (v/v) paraformaldehyde and 1.25% (v/v) glutaraldehyde in 0.05 M PIPES buffer, pH 7.2. The samples were dehydrated with a graded ethanol series and embedded in London Resin White (LR White, Electron Microscopy Sciences, Fort Washington, PA, USA) acrylic resin. The antibody used (raised in rabbit) was against the P subunit of mitochondrial glycine decarboxylase (GDC) IgG from *Pisum sativum* L. (courtesy of Dr David Oliver). Preimmune serum was used as a control.

For transmission electron microscopy immunolabeling, thin sections (~70–90 nm) on Formvar-coated nickel grids were incubated for 1 h in TBST+BSA to block non-specific protein binding on the sections. They were then incubated for 3 h with either the preimmune serum diluted in TBST+BSA or anti-GDC (1:10) antibodies. After washing with TBST+BSA, the sections were incubated for 1 h with Protein A-gold (15 nm) diluted 1:100 with TBST+BSA. The sections were washed sequentially with TBST+BSA, and TBST and distilled water, and then post-stained with a 1:3 dilution of 0.5% (w/v) potassium permanganate and 2% (w/v) uranyl acetate. Images were collected using a FEI Tecnai G2 transmission electron microscope. The density of labeling was determined by counting the gold particles on electron micrographs and calculating the number per unit area (µm^2^) in the mitochondria, versus background labeling density in the rest of the cell. For each cell type, replicate measurements were made on parts of cell sections (*n*=10–60 images from at least two different experiments).

### Western blot analysis

Total proteins were extracted from leaves by homogenizing 0.2 g of tissue in 0.2 ml of extraction buffer (100 mM Tris-HCl, pH 7.5, 10 mM (w/v) MgCl_2_, 1 mM (w/v) EDTA, 15mM (v/v) β-mercaptoethanol, 20% (v/v) glycerol and 1mM phenylmethylsulfonyl fluoride). Extraction was continued by adding 0.3 ml 60 mM Tris-HCl, pH 7.5, 4% (w/v) SDS, 20% (v/v) glycerol, 0.5% (v/v) β-mercaptoethanol and 0.1% (w/v) bromphenol blue. After boiling for 5 min for SDS-PAGE the supernatant was collected after centrifugation at 14000 ×*g* for 5 min and protein concentration was determined with an RCDC protein quantification kit (Bio-Rad, Hercules, CA, USA), which tolerates detergents and reducing agents. Protein samples (20 µg) were separated by 10% SDS-PAGE, blotted onto nitrocellulose, and probed with anti-*Amaranthus hypochondriacus* NAD-ME IgG which was prepared against the 65 KDa α subunit, courtesy of J. Berry (Long and Berry, 1996) (1:2000), anti-*Zea mays* 62 KDa NADP-ME IgG, courtesy of C. Andreo (Maurino *et al.*, 1996) (1:2500), commercially available anti-*Zea mays* PEPC IgG (1:100 000) (Chemicon, Temecula, CA, USA), anti-*Zea mays* pyruvate, Pi dikinase (PPDK) IgG, courtesy of T. Sugiyama (1:50 000), overnight at 4 ºC. Goat anti-rabbit IgG-alkaline phosphatase conjugate antibody (Bio-Rad) was used at a dilution of 1:20000 for detection. Bound antibodies were localized by developing the blots with 20 mM nitroblue tetrazolium and 75 mM 5-bromo-4-chloro-3-indolyl phosphate in detection buffer (100 mM Tris-HCl, pH 9.5, 100 mM NaCl, 5 mM MgCl_2_). The intensities of bands in western blots were quantified with an image analysis program (ImageJ 1.37v) and expressed relative to mean level in seven C_4_ species, which was set at 100%.

### Measurements of rates of photosynthesis

Gas exchange was measured using the LI-6400XT portable photosynthesis system equipped with broadleaf chamber (LI-6400-02B) in response to varying CO_2_ at 1000 µmol quanta m^−2^ s^−1^ PPFD and 25 ^○^C and in response to varying light intensity at 400 µbar CO_2_ and 25 ^○^C. Measurements were performed in greenhouse with enclosing of one leaf or leaf portion inside of chamber. For each experiment the leaves were illuminated with 1000 PPFD under 400 µbar CO_2_ and 25 ^○^C until a steady state rate of CO_2_ fixation was obtained (generally 30 min). For varying CO_2_ experiments the CO_2_ level was decreased, and then increased up to 2000 µbar at 5 min intervals. For varying light experiments, measurements were made beginning at 2000 PPFD, with decreasing levels at 4 min intervals. The CO_2_ compensation point (Г) was determined by extrapolating the initial slope of CO_2_ response curve through the *x*-axis and taking the zero intercept. The leaf area was calculated from digital image of the leaf portion enclosed in the chamber, using an image analysis program (ImageJ 1.37v).

### δ^13^C values

δ^13^C values, a measure of the carbon isotope composition, were determined at Washington State University on leaf samples taken from plants using a standard procedure relative to PDB (Pee Dee Belemnite) limestone as the carbon isotope standard (Bender *et al.*, 1973). Plant samples were dried at 80 °C for 24 h, milled to a fine powder and then 1–2 mg were placed into a tin capsule and combusted in a Eurovector elemental analyzer. The resulting N_2_ and CO_2_ gases were separated by gas chromatography and admitted into the inlet of a Micromass Isoprime isotope ratio mass spectrometer (IRMS) for determination of ^13^C/^12^C ratios. δ^13^C values were calculated where δ^13^C=1000×(R_sample_/R_standard_)–1, where R=^13^C/^12^C.

### C_4_ biochemical-type evolution

A combined data matrix of chloroplast and nuclear DNA sequences was prepared from the data used by Ocampo and Columbus (2010, 2012) and Ocampo *et al.* (2013) to obtain an evolutionary framework to estimate C_4_ variant diversification within Portulacaceae. Sampling included species of *Portulaca* with known C_4_ biochemical types. Sequences were aligned using MUSCLE version 3.7 (Edgar, 2004), followed by manual alignment in MEGA version 7.0.14 (Kumar *et al.*, 2016). The combined data matrix was partitioned by locus and analyzed under maximum likelihood (ML; Felsenstein, 1973) in RA×ML version 7.2.6 (Stamatakis, 2006) under the GTRGAMMA model. Clade support was calculated by nonparametric bootstrapping (Felsenstein, 1985) from 10000 replicates performed simultaneously with the ML search using the ‘-f a’ option. The phylogenetic analysis was executed using the High Performance Computing Cluster at the California Academy of Sciences. Evolution of C_4_ biochemical types was estimated by ML over the ML tree using Mesquite version 3.04 (Maddison and Maddison, 2016) under the Markov k-state 1 parameter model (Lewis, 2001).

## Results

### Carbon isotope composition of leaves

The carbon isotope values of leaves of the three *Portulaca* species in the Crytopetala clade, *P. cryptopetala*, *P. hirsutissima* and *P. mucronata*, were all C_3_-like (δ^13^C −26.6 to −28.6^o^/_oo_). In all species in the Pilosa clade that were analyzed the values were in the range of C_4_ plants (−12.1 to −17.4^o^/_oo_) (Table 1).

### General view of plants and leaf structure

**Fig. 1.**
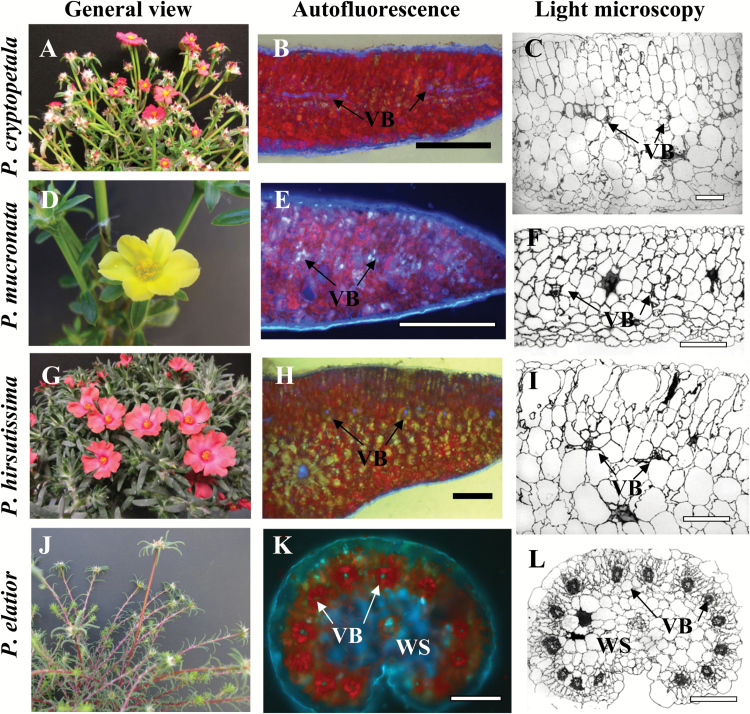
General view of *Portulaca* species (left panels, A, D, G, J), distribution of chlorenchyma under the fluorescent microscope (middle panels, B, E, H, K), and light microscopy of leaf cross sections (right panels, C, F, I, L). *P. cryptopetala* (B, C)*, P. mucronata* (E, F) and *P. hirsutissima* (H, I), all have C_3_ like dorsoventral type leaf anatomy. In *P. cryptopetala* and *P. mucronata* (B, C, E, F), all vascular bundles (VB), including the main vein, are distributed in the median paradermal plane; red fluorescence from chlorophyll is distributed more or less evenly through the leaf mesophyll (B, E). In *P. hirsutissima* (H, I), VB are positioned close to the adaxial side while the main vein is in the center of the leaf below lateral veins; red chlorophyll fluorescence is concentrated in the adaxial palisade mesophyll layers with water storage (WS) spongy mesophyll cells in the middle of the leaf (H). *P. elatior* has Pilosoid type of anatomy (K, L) with peripheral distribution of VB with each vein surrounded by two Kranz chlorenchyma layers; WS tissue is around the main vein. VB, vascular bundles; WS, water storage tissue. Scale bars: 1 mm for B; 200 µm for C, F, I, L; 0.5 mm for E, H, K.

Figure 1 shows the general view of representative *Portulaca* species that were analyzed in the most detail in this study. Two of the species, *P. cryptopetala* (Fig. 1A) and *P. mucronata* (Fig. 1D) have flattened leaves, while *P. hirsutissima* (Fig. 1G) has thicker and more succulent terete (terete means succulent circular or distorted circle shape) leaves. All three species clearly have C_3_-like dorsoventral type of anatomy with vascular bundles (VB) situated under the palisade layers, in the medium part of leaf lamina between palisade and spongy parenchyma (Fig. 1B, C, E, F, H, I). In two species, *P. cryptopetala* and *P. mucronata* there are two layers of palisade parenchyma on the adaxial side and three to four layers of spongy parenchyma cells on the abaxial side; the VB are surrounded by BS cells (Fig. 1C, F). The general leaf anatomy in *P. hirsutissima* (Fig. 1I) is similar except for the intensive development of spongy water storage (WS) parenchyma in the middle part of the leaf (Fig. 1I). In all three species the BS cells around lateral VB are rather large; but, their size is similar to the surrounding M cells (Fig. 1C, F, I).

In *P. cryptopetala* and *P. mucronata*, the main vein is located in the same plane as all lateral VBs (not shown); however; in *P. hirsutissima* the main vein is well below the lateral veins and it is surrounded by spongy WS parenchyma (Fig. 1H).

Representative species from the Pilosa clade (*P. biloba, P.* cf. *gilliesii, P. elatior, P. halimoides, P. smallii* and *P. suffrutescens*) have lanceolate semi-terete or cylindrical fleshy leaves with Kranz anatomy of Pilosoid type. As illustrated with *P. elatior* (Fig. 1K, L), in this type of anatomy the VB are distributed around the leaf periphery with each individual vein surrounded by two specialized chlorenchyma layers characteristic of Kranz-type anatomy. The main vein is located more or less in the center of the leaf and is surrounded by WS tissue.

### Transmission electron microscopy

**Fig. 2.**
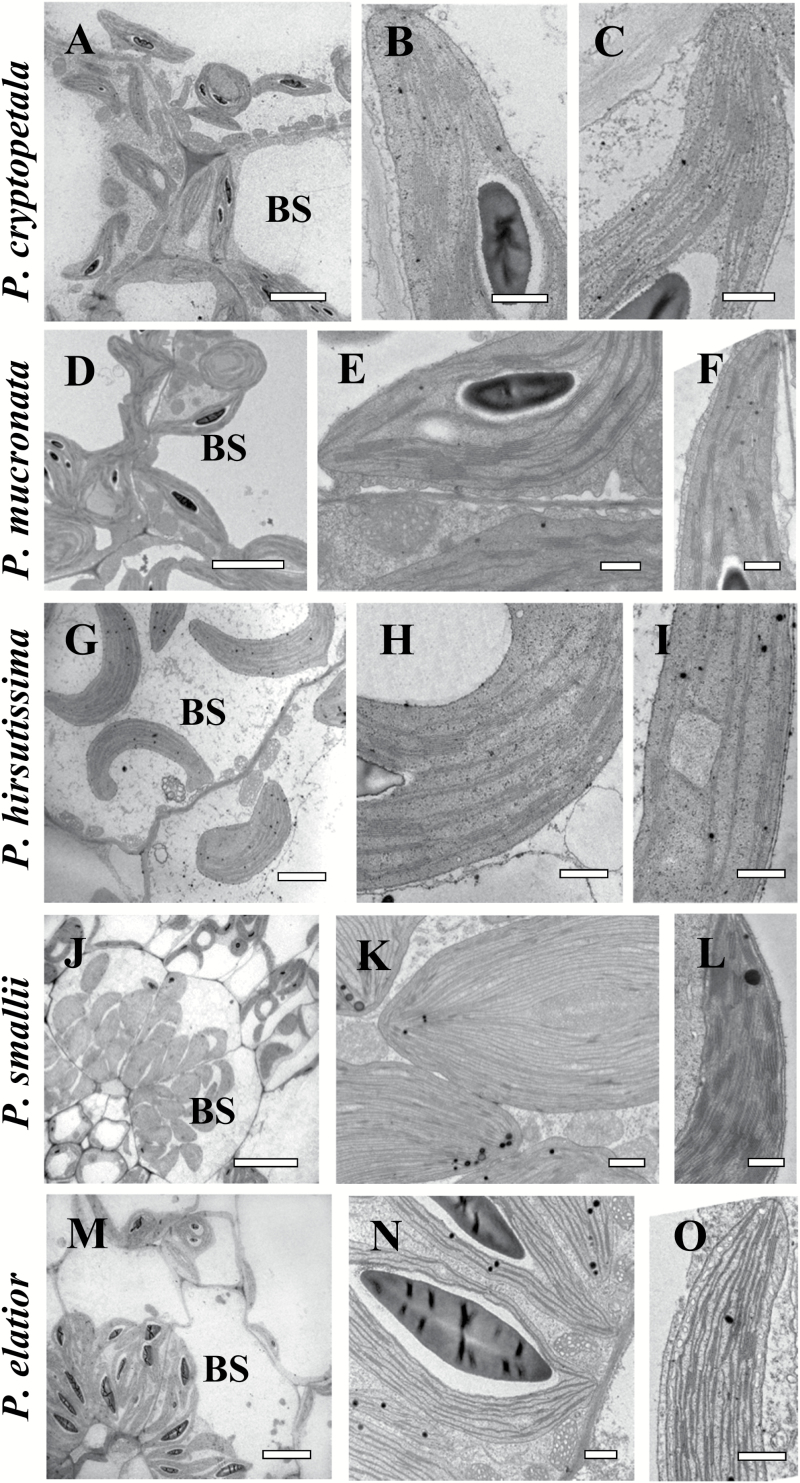
Electron microscopy of bundle sheath (BS) cells (A, D, G, J, M), and chloroplasts in BS (B, E, H, K, N) and mesophyll (M) (C, F, I, L, O) cells in representative *Portulaca* species. *P. cryptopetala* (A–C)*, P. mucronata* (D–F), *P. hirsutissima* (G–I)*, P. smallii* (J–L), and *P. elatior* (M–O). Panels (A, D, G, J, M) show the distribution of organelles in BS cells in centripetal position. In panels (B, E, H, N) the BS chloroplasts have well-developed grana, while BS chloroplasts in *P. smallii* (K) are grana-deficient. In contrast to the developed grana in M chloroplasts in (C, F, I and L), panel O shows grana-deficient M chloroplasts in *P. elatior*. Panel N illustrates occurrence of mitochondria around BS chloroplasts. BS, bundle sheath; M, mesophyll;. Scale bars: 5 µm for A, D, G, M; 20 µm for J; 1 µm for K; 0.5 µm for B, C, E, F, H, I, L, N, O.

In three species, *P. cryptopetala, P. hirsutissima* and *P. mucronata*, the BS cells contain a significant number of organelles in the centripetal position, adjacent to VB and along the radial cell walls (Fig. 2A, D, G). M and BS chloroplasts in all three species have a similar level of grana development with medium-sized grana (Fig. 2B, C, E, F, H, I). BS cells contain numerous centripetally arranged enlarged mitochondria (Fig. 2A, D, G).

In all six species with Pilosoid Kranz-type leaf anatomy, the BS cells surrounding the minor veins contain numerous organelles in a centripetal position (illustrated in *P. smallii* and *P. elatior,*Fig. 2J, M). A more detailed study of M and BS ultrastructure revealed significant differences. In five species (*P. biloba, P.* cf. *gilliesii, P. halimoides, P. smallii* and *P. suffrutescens*), the BS cells contain nearly agranal chloroplasts while M chloroplasts have rather well-developed grana as shown for *P. smallii* in Fig. 2K, L. However, one species, *P. elatior*, has a reverse type of chloroplast ultrastructure; the chloroplasts in the BS cells have numerous mid-sized grana (Fig. 2N) while the M chloroplasts have a more extensive single thylakoid system (Fig. 2O). This species also has numerous large mitochondria with specific tubular cristae in BS cells (Fig. 2N).

### Vein density

**Fig. 3.**
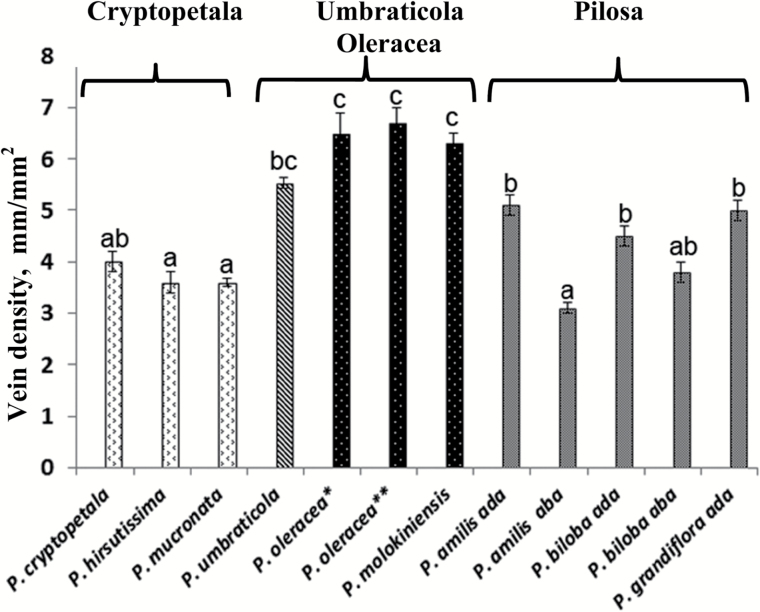
Graph showing the density of venation in representative *Portulaca* species. For two species, *P. amilis* and *P. biloba*, vein density is shown for both sides of the leaf, adaxial and abaxial (ada and aba on graph). For *P. oleracea*, two accessions were studied: *, seeds from Pullman, WA, USA; and **, seeds from USDA PI 0121921. *n*≥10. Different letters indicate significant differences between species and leaf sides, *P*≤0.05.

The vein density in the three species in the Cryptopetala clade (*P. cryptopetala, P. hirsutissima* and *P. mucronata*) which lack Kranz anatomy was analyzed in comparison to Kranz-type species including *P. umbraticola* with Atriplicoid leaf anatomy from Umbraticola clade, two species in the Oleracea clade with modified Atriplicoid-type anatomy (*P. molokiniensis* and two varieties of *P. oleracea*) and three species in the Pilosa clade with Pilosoid-type anatomy (*P. amilis*, *P. biloba* and *P. grandiflora*) (Fig. 3). Among these, in general species in the Cryptopetala clade had the lowest vein density while the highest vein densities were in the two species in the Oleracea clade (vein density ~1.8-fold higher than the Cryptopetala species). In the species in the Pilosa clade, the vein densities on the adaxial side of leaves were on average ~1.3-fold higher than the Cryptopetala species. *Portulaca umbraticola* had high vein density, appearing to be intermediate between Pilosa and Oleracea clades. The three species with Pilosoid Kranz-type leaf anatomy showed similar vein densities on the adaxial leaf side while the densities in the two species with flattened leaves (*P. amilis* and *P. biloba*) were 1.2 and 1.7 times lower on the abaxial leaf side.

### Cotyledon anatomy

**Fig. 4.**
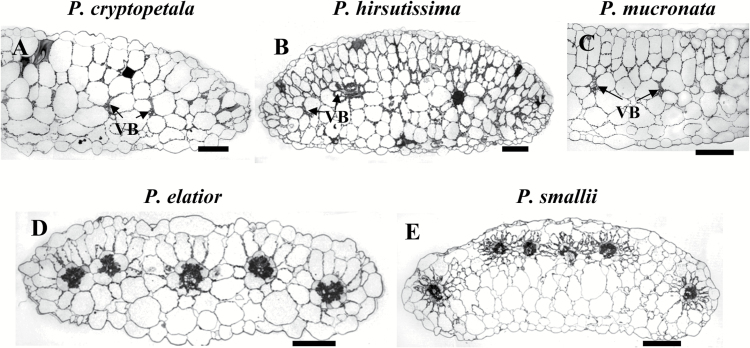
Light micrographs showing cross sections of cotyledons for five *Portulaca* species. Three species have dorsoventral cotyledon anatomy: *P. cryptopetala* (A), *P. hirsutissima* (B), *P. mucronata* (C). Note the enlarged number of organelles in bundle sheath cells of these species (most obvious in *P. mucronata*). *P. elatior* (D) and *P. smallii* (E) have cotyledons with Atriplicoid Kranz-type anatomy. VB, vascular bundle. Scale bars, 200 µm.

The species in the Cryptopetala clade, *P. cryptopetala, P. hirsutissima* and *P. mucronata*, have cotyledons with C_3_-like dorsoventral anatomy, with one to two layers of palisade-like M cells on the adaxial side and several layers of spongy M cells towards the abaxial side (Fig. 4A–C). The number of spongy parenchyma layers varies between species: from one or two layers in *P. cryptopetala* and *P. mucronata* to mostly three in semi-terete cotyledons of *P. hirsutissima* with a little thicker mid-section. VB are distributed in a lateral plane in the central part of the lamina in *P. cryptopetala* and *P. mucronata* (Fig. 4A, C) and they are located closer to the adaxial side of the lamina under the palisade parenchyma in *P. hirsutissima* (Fig. 4B). In all three species the VB are surrounded by rather large BS cells with a higher density of organelles (chloroplasts and mitochondria) in the centripetal position or along the radial cell walls, similar to that described for leaves (structural analysis by TEM is not shown).

The cotyledons in five studied species in the Pilosa clade with Kranz-type anatomy in leaves (*P. biloba, P. elatior, P. halimoides, P. smallii* and *P. suffrutescens*) all have Atriplicoid-like Kranz anatomy with VB distributed in one lateral plane (illustrated only for *P. elatior* and *P. smallii,*Fig. 4D, E). The VB surrounded by two layers of Kranz chlorenchyma are located under the adaxial epidermis, with variable number of spongy parenchyma layers towards the abaxial epidermis, from one layer in *P. elatior* to mostly two layers in *P. biloba, P. halimoides* and *P. suffrutescens*. In *P. smallii* the VB are located closer to the adaxial side of the lamina with three/four layers of spongy parenchyma cells on the adaxial side of the cotyledon (Fig. 4E).

### Immunolabeling for GDC

**Fig. 5.**
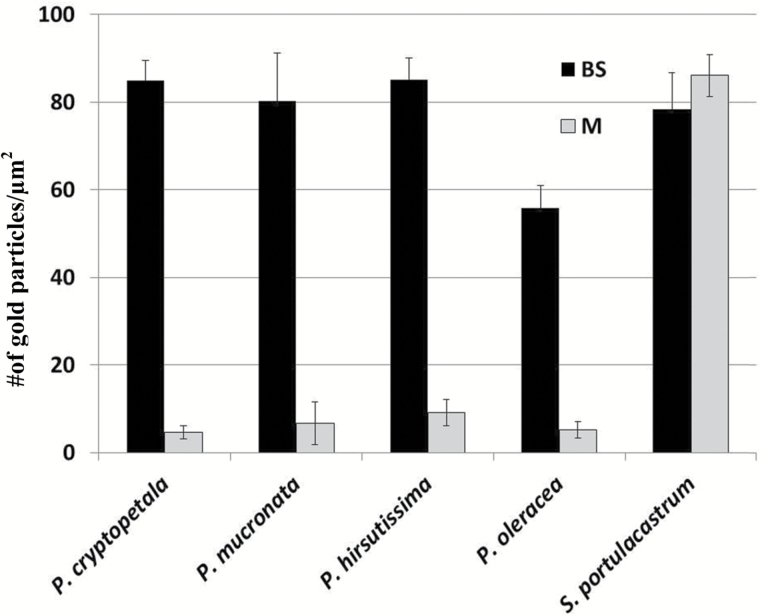
Density of immunogold labeling of GDC in mitochondria of mesophyll (M) and boundle sheath (BS) cells of four species of *Portulaca* and *Sesuvium portulacastrum* as C_3_ control. Shown is the number of gold particles per μm^2^ of mitochondria. *n*≥10.

In situ immunolabeling for GDC was performed for *P. cryptopetala, P. hirsutissima* and *P. mucronata* in the Cryptopetala clade in comparison to the C_4_ species *P. oleracea* (Fig. 5; Supplementary Fig. S1 at *JXB* online). Analysis of the density of immunogold particles for anti-GDC antibody shows strong selective localization in BS mitochondria (~10-fold higher than in M mitochondria) in *P. oleracea*. Similarly, in all three species, *P. cryptopetala, P. hirsutissima* and *P. mucronata,* there is clear selective labeling for GDC in BS mitochondria (Fig. 5). Supplementary Fig. S1 shows electron microscopy images of the immunolabeling for anti-GDC antibody, where it is selectively localized in BS mitochondria in *P. oleracea* and the C_3_-C_4_ intermediates, while in the C_3_ species *Sesuvium portulacastrum* there is similar density of labeling in mitochondria in M and BS cells (Fig. 5).

### Western blot analysis

**Fig. 6.**
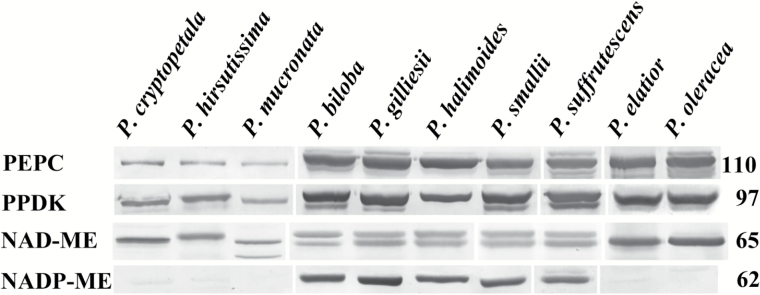
Western blots for four C_4_ pathway enzymes from total proteins extracted from leaves of ten *Portulaca* species. Blots were probed with antibodies raised against PEPC, PPDK, NAD-ME and NADP-ME. Numbers at the right indicate molecular mass in kilodaltons. The originals were modified to group species according to the presentation (vertical lines); there were no selective changes in positions or densities of bands on the membrane. The reason for labeling of two bands in some species with the antibody for NAD-ME (prepared against *A. hypochondriacus* α-NAD-ME) is not known. It may represent the expression of two isoforms of the α-NAD-ME, or the antibody may have low reactivity with the smaller subunit β-NAD-ME in some species (see Murata *et al.*, 1989; Tronconi *et al.*, 2008; Maier *et al.*, 2011).

Immunoblots are shown for photosynthetic enzymes of the C_4_ cycle, PEPC, PPDK, NAD-ME and NADP-ME, from total proteins extracted from leaves of *Portulaca* species (Fig. 6). In the three species in the Cryptopetala clade, (*P. cryptopetala*, *P. hirsutissima* and *P. mucronata*) the labeling for C_4_ cycle enzymes PEPC and PPDK was much lower than in the other species all of which have Kranz anatomy. Analyses of the relative band densities (measured by Image J) showed the density of labeling in the intermediates for PEPC was 7–15% and for PPDK was 14–20% compared to the mean value for the seven C_4_ species. The three species from the Cryptopetala clade had lower labeling for NAD-ME-type enzyme compared to the NAD-ME species *P. elatior* and *P. oleracea*, and no detectable labeling for NADP-ME (Fig. 6). Among the other species, five are in the Pilosa clade (*P. biloba, P.* cf. *gilliesii, P. elatior, P. halimoides, P. smallii* and *P. suffrutescens*), while the other, *P. oleracea*, is in the Oleracea clade. With respect to C_4_ decarboxylases, all species from the Pilosa clade have high labeling for NADP-ME with the exception of *P. elatior*; it has high labeling for NAD-ME and no detectable labeling for NADP-ME, which is similar to *P. oleracea.*

### Gas exchange analysis

**Fig. 7.**
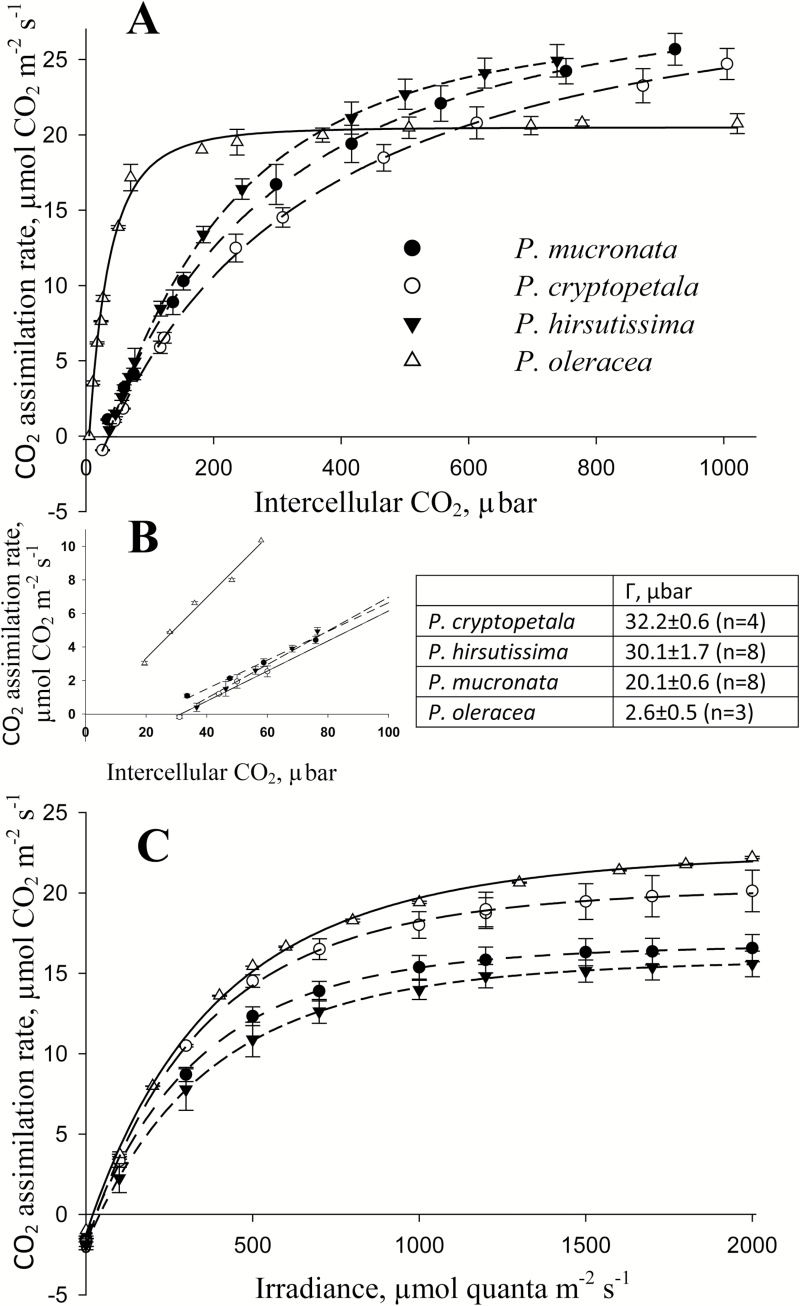
Gas exchange analyses of four *Portulaca* species, *P. cryptopetala, P. hirsutissima, P. mucronata, P. oleracea.* (A) Rates of CO_2_ fixation in response to varying intercellular levels of CO_2_ at 21% O_2_, 25 ºC and 1 000 PPFD. (B) CO_2_ compensation point (Г) measured at 1 000 PPFD, 21% O_2_ and 25 ºC. (C) Rates of CO_2_ fixation in response to varying light intensities at 21% O_2_, 25 ºC and 400 µbar CO_2_. For the CO_2_ response curves (panel A), the results represent the average of 3–5 replications (for each replication, mean values were taken from CO_2_ response curves measured from high to low, followed by low to high levels of CO_2_). In panel C, the results represent the average of 2–4 replications from measurements made on different leaves (averages were taken from results obtained with changes from high to low light intensity).

Gas exchange analyses were made to compare photosynthetic features of species in the Cryptopetala clade with a representative C_4_ species, *P. oleracea*. The response of photosynthesis to varying intercellular levels of CO_2_ were measured under 1000 PPFD, 25 ºC and atmospheric O_2_ (21%). With increasing levels of CO_2_, there was a rapid increase in photosynthesis in *P. oleracea* with near saturation at ~200 μbar CO_2_, whereas *P. cryptopetala*, *P. hirsutissima* and *P. mucronata* show strong a continual increase in photosynthesis up to 800–1000 µbar CO_2_ with very similar rates under high CO_2_ (Fig. 7A). When photosynthesis was measured under near current atmospheric levels of CO_2_, 400 μbar (with resulting C_i_ values ~200–250 µbar), the rate of photosynthesis was higher in *P. oleracea* than in the other three species. The CO_2_ compensation points (Γ) determined from extrapolation of the initial slopes of the response curve, was 2.6 μbar for *P. oleracea*, while values in the Cryptopetala species *P. cryptopetala* and *P. hirsutissima* were ~ 30 μbar, and in *P. mucronata* Γ was 20 μbar (Fig. 7B). In light response curves, at 400 µbar CO_2_ and 25 ºC, the response of photosynthesis was similar among the species (Fig. 7C). In the Cryptopetala species photosynthesis reaches saturated rates at ~1100 PPFD while there was some marginal increase in rates in *P. oleracea* up to 2000 PPFD. The maximum rates at 2000 PPFD in *P. hirsutissima* and *P. mucronata* were similar, and significantly (*P*<0.05) lower than in *P. cryptopetala* and *P. oleracea* (Fig. 7C).

### Evolution of C_4_ biochemical types

**Fig. 8.**
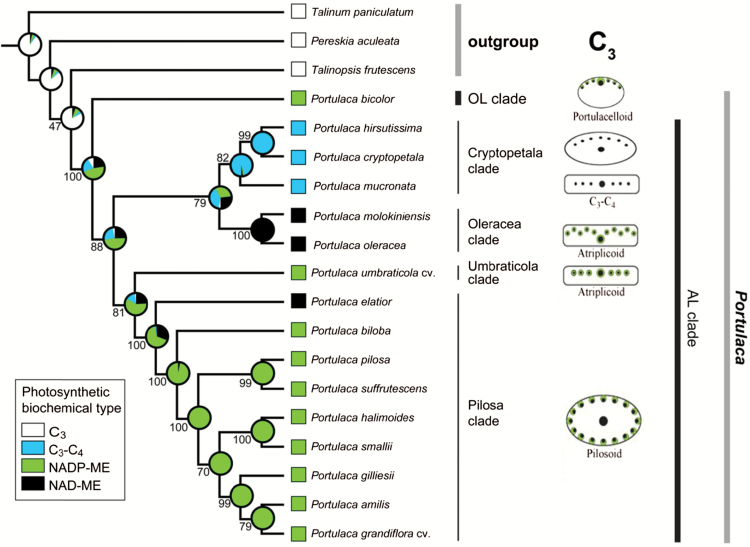
Maximum likelihood (ML) estimation of C_4_ biochemical variants and C_3_-C_4_ intermediacy evolution in *Portulaca*. Proportional likelihoods in the form of a pie chart are shown at each node of the ML reconstruction. Numbers below or above the branches are bootstrap support values derived from a RA×ML analysis. Schematic drawings for illustration of leaf anatomical types are adapted from Ocampo *et al.* (2013).

The sampling for the phylogenetic analysis included 16 *Portulaca* species with known C_4_ biochemical types (see Voznesenskaya *et al.*, 2010; Ocampo *et al.*, 2013; this study) and three representatives of close-related taxa as outgroup (Table 2). Relationships within *Portulaca* follow those obtained by Ocampo and Columbus (2012), where the genus and the relations among major clades are well supported (Fig. 8). The analysis shows the NADP-ME biochemical variant is the ancestral C_4_ type for *Portulaca*. It predicts that there were two independent switches to the NAD-ME type, one in the ancestor of the Oleracea clade and one in *P. elatior* (earliest divergent lineage of the Pilosa clade). The C_3_-C_4_ condition originated only once in the ancestor of the Cryptopetala clade.

**Table 2.** GenBank accession numbers for samples used in the phylogenetic analysis. -, not available.

Taxon	ITS	*ndhF*	*trnT-psbD* intergenic spacer	*ndhA* intron
***Portulaca***				
*P. amilis* Speg.	JF508527	JF508674	JF508757	HQ241593
*P. bicolor* F. Muell.	JF508532	JF508679	JF508762	HQ241594
*P. biloba* Urb.	JF508533	JF508680	JF508763	JF508613
*P. cryptopetala* Speg.	JF508538	JF508685	JF508768	HQ241596
*P. elatior* Mart. ex Rohrb.	JF508542	JF508689	JF508772	HQ241598
*P. gilliesii* Hook.	JF508548	JF508695	JF508778	JF508624
*P. grandiflora* Hook. cv.	JF508550	JF508697	JF508780	JF508626
*P. halimoides* L.	JF508552	JF508699	JF508782	JF508627
*P. hirsutissima* Cambess.	KC690150	-	-	-
*P. molokiniensis* Hobdy	JF508562	JF508709	JF508792	HQ241602
*P. mucronata* Link	KC690151	-	-	-
*P. oleracea* L.	JF508566	JF508713	JF508796	JF508638
*P. pilosa* L.	JF508585	JF508732	JF508815	HQ241603
*P. smallii* P. Wilson	JF508595	JF508742	JF508825	JF508663
*P. suffrutescens* Engelm.	JF508597	JF508744	JF508827	JF508665
*P. umbraticola* Kunth cv. ‘wildfire mixed’	JF508600	JF508747	JF508830	JF508668
**Outgroup**				
*Pereskia aculeata* Mill.	JF508526	JF508673	JF508756	HQ241587
*Talinopsis frutescens* A. Gray	JF508607	JF508754	JF508837	HQ241613
*Talinum paniculatum* (Jacq.) Gaertn.	JF508608	JF508755	JF508838	HQ241618

## Discussion

In this study on species which belong to the AL clade of *Portulaca*, the focus was on clades Pilosa and Cryptopetala. *Portulaca biloba, P.* cf. *gilliesii, P. elatior, P. halimoides, P. smallii* and *P. suffrutescens* are from the Pilosa clade, and they all have C_4_-type carbon isotope ratios (this study; Ocampo *et al.*, 2013). The discovery that this clade has not only NADP-ME species, but also an NAD-ME type C_4_ (*P. elatior*, which is widely distributed from the Caribbean to Venezuela and Brazil) provides insight into diversity in evolution of forms of C_4_ in the genus. The Cryptopetala clade, which is composed of *P. cryptopetala, P. hirsutissima* and *P. mucronata*, is the only lineage that has species with C_3_-type carbon isotope ratios (Table 1, Ocampo *et al.*, 2013); in this study all three species are shown to be C_3_-C_4_ intermediates.

### Evolutionary trends in anatomy

#### Leaf anatomy

Among the three intermediate *Portulaca* species in the Cryptopetala clade, *P. cryptopetala* and *P. mucronata* are sister species, while *P. hirsutissima* is an early-divergent (see Fig. 8). The former two species have rather succulent, flattened leaves, while *P. hirsutissima* has terete leaves with a thicker central part. This is consistent with differences in structure: *P. cryptopetala* and *P. mucronata* have broad leaves with VB distributed in one plane, while *P. hirsutissima* differs in having semi-terete leaves with all lateral VBs distributed under the adaxial epidermis and the main vein underneath, surrounded by WS. In *P. cryptopetala* and *P. mucronata* the distribution of VB is similar to the C_4_ species in the Umbraticola clade having Atriplicoid Kranz-anatomy, while in *P. hirsutissima* the leaf anatomy resembles the Portulacelloid-type Kranz-anatomy found in the Australian clade C_4_ species (see Fig. 8 for anatomical types).

Most of the C_3_-C_4_ intermediate species found in different dicot lineages have a general leaf structural pattern that resembles closely related C_4_ species. Most of them have flattened dorsoventral or isopalisade leaves with enlarged BS cells. This type of anatomy in flattened leaves is characteristic for C_3_-C_4_ intermediates in genera *Flaveria* (Holaday *et al.*, 1984*b*) and *Parthenium* (Moore *et al.*, 1987) in family Asteraceae, in *Euphorbia* in family Euphorbiaceae (Sage *et al.*, 2011), *Heliotropium* in family Boraginaceae (Muhaidat *et al.*, 2011), and *Alternantera* in family Amaranthaceae (Rajendrudu *et al.*, 1986). They have Kranz-like Atriplicoid-type anatomy as the most advanced stage of evolution from intermediates towards C_4_. This includes several species with C_4_-like photosynthesis (*Flaveria brownii,*Cheng *et al.*, 1988) and two intermediate *Heliotropium* species with Kranz-like anatomy (Muhaidat *et al.*, 2011). Genera *Moricandia* (Apel and Ohle, 1979; Holaday and Chollet, 1984*a*) and *Diplotaxis* (Ueno *et al.*, 2003) also have intermediate species with similar type of anatomy in flattened leaves; but there are no known C_4_ relatives in family Brassicaceae. In family Cleomaceae, the С_3_-С_4_ intermediate *Cleome paradoxa* has flattened leaves with isopalisade anatomy with all VB distributed in one plane, Atriplicoid-like (Voznesenskaya *et al.*, 2007). It was suggested to be closer phylogenetically to C_4_*C. angustifolia* which has Glossocardioid Kranz-type leaf anatomy; nevertheless, the phylogenetic position of this species is not far from C_4_*Cleome* (*Gynandropsis*) *gynandra* with Atriplicoid-type leaf anatomy (Feodorova *et al.*, 2010).

In considering intermediates and forms of Kranz in families Chenopodiaceae and Portulacaceae, there is large diversity in C_4_ from Atriplicoid type to forms of Kranz with VB distributed around WS tissue in succulent leaves. In Chenopodiaceae, the type of leaf anatomy in the C_3_-C_4_ intermediate *Sedobassia sedoides* resembles the Kranz Kochioid type in C_4_*Bassia* in the positioning of veins with respect to WS tissue and the positioning of BS cells with respect to VB where they are distributed only on the outer side of the lateral VB (Freitag and Kadereit, 2014). Also, Kranz-like Sympegmoid and Kranz-like Salsoloid are C_3_-C_4_ intermediate types of leaf anatomy found in *Salsola* s.l. species, which resemble Kranz Salsoloid-type in peripheral distribution of M and positioning of minor veins in a circular pattern around WS tissue in succulent leaves (Voznesenskaya *et al.*, 2013; Schüßler *et al.*, in press). In the present study, the leaf anatomy in C_3_-C_4_ intermediate *Portulaca* species follow this trend in showing Atriplicoid-like and Portulacelloid-like types of anatomy with related C_4_ species having these forms of Kranz anatomy.

#### Cotyledon anatomy

The three Cryptopetala species, which are shown to have C_3_-C_4_ intermediate type of anatomy in leaves, all have a similar dorsoventral type of anatomy in cotyledons with centripetally arranged organelles in BS cells. In one species, *P. hirsutissima*, the VB are distributed close to the adaxial epidermis, with greater number of WS spongy parenchyma layers towards the abaxial side, compared to other species with VB distributed in the central lateral plane of the lamina. In closely related families Cleomaceae and Brassicaceae, the C_3_-C_4_ intermediates *Cleome paradoxa* and *Moricandia arvensis* also have cotyledons with C_3_-C_4_ intermediate anatomy (Rylott *et al.*, 1998; Koteyeva *et al.*, 2011). In other intermediate species (e.g. *Salsola* and *Sedobassia sedoides*, family Chenopodiaceae), the cotyledons have C_3_-type anatomy (Voznesenskaya, Koteyeva, unpublished data). In species where the cotyledons also have intermediate phenotype, this could be advantageous in climates where seedlings are more prone to losses due to photorespiration (warmer climates). In general, intermediate species having C_3_- versus C_3_-C_4_-type photosynthesis in cotyledons may be an example of heterobathmy (Takhtajan, 1959), a phenomenon which results in unequal levels of specialization of different parts of one organism or taxon, achieved during the process of biological evolution.

In five species analyzed in the Pilosa clade, which have Pilosoid Kranz-type in leaves, the cotyledons all have Atriplicoid-like Kranz anatomy with some differences in shape and number of WS spongy layers on the abaxial side. Thus cotyledons in *P. elatior*, *P. biloba, P. halimoides* and *P. suffrutescens* have classical Atriplicoid-type anatomy with all Kranz-units (VB surrounded by two layers of chlorenchyma, characteristic for C_4_ species, see Peter and Katinas, 2003) distributed in a central plane and one or two layers of spongy parenchyma on the adaxial side. However, in cotyledons of *P. smallii*, all Kranz-units are distributed under the adaxial epidermis with several layers of WS spongy parenchyma underneath. It is easy to imagine that this type of structure could represent a transitional step between flat-leaved cotyledons with Atriplicoid anatomy to the Pilosoid anatomy characteristic of that in leaves of *P. smallii*. In a previous study, *P. amilis* and *P. grandiflora* in the Pilosa clade, *P. umbraticola* in the Umbraticola clade and *P. oleracea* in the Oleracea clade were shown to have Atriplicoid-like anatomy in cotyledons; however, *P.* cf. *bicolor* in the OL clade, has, as in leaves, Portulacelloid type anatomy in the cotyledons (Voznesenskaya *et al.*, 2010). In general, all *Portulaca* species examined having Kranz-type anatomy in leaves, also have Kranz anatomy in the cotyledons. As noted by Voznesenskaya *et al.* (2010), in C_4_ dicots that have been analyzed in other families, the anatomy and type of photosynthesis in cotyledons has often been found to be similar to leaves. However, there is diversity with some *Kochia* species having Kochioid-type anatomy in leaves and Atriplicoid-type anatomy in cotyledons and with some *Salsola* species having Salsoloid-type anatomy in leaves with Atriplicoid-type anatomy in cotyledons (Pyankov *et al.*, 1999*a*, 2000); and some chenopods having Kranz anatomy and C_4_ photosynthesis in leaves, with cotyledons having C_3_-type anatomy and biochemistry (Pyankov *et al.*, 1999*b*, 2000; Voznesenskaya *et al.*, 1999; Lauterbach *et al.*, 2016). These variations are of interest in considering genetic factors that control differences in forms of anatomy and biochemistry that develop in C_4_ dicot leaves versus cotyledons.

### Trends in venation density in leaves

Adaptation of plants to arid environments is often accompanied by an increase in vein density; at the same time it was shown that in most cases vein density decreased with increasing succulence, which is the second structural and/or functional way of plant adaptation to aridity (Ogburn and Edwards, 2013). Both forms of adaptation likely occurred in evolution of types of Kranz anatomy in C_4_ species of families Chenopodiaceae and Portulacaceae. Together with the tendency to decrease leaf surface area to volume ratio, most succulents have subterete to terete leaves (more or less roundish in cross section). This leaf shape is often accompanied by development of a 3D type of venation. This pattern helps to maintain the most balanced hydraulic pathway to support photosynthesis by a closer position of veins to chlorenchyma, with a decrease in the path from the vein to leaf surface in thick succulent leaves (Ogburn and Edwards, 2009, 2013). Increased vein density is considered to be one of the crucial factors in the evolution of C_4_ photosynthesis in many dicot and grass lineages (Sage *et al.*, 2012). However, studies of venation in representative *Salsola* species with different types of photosynthesis showed there is no significant difference between C_3_, C_3_-C_4_ intermediates and C_4_ species in vein density, while there is a clear increase in volume of WS tissue (Voznesenskaya *et al.*, 2013).

All *Portulaca* species have succulent leaves that differ in thickness, shape and type of anatomy. In C_4_*Portulaca* lineages there are differences in vein density along with variation in the pattern of vein distribution in different anatomical types, with the highest density in species having Atriplicoid anatomy.

In intermediate *Portulaca* species, the vein density is about 2–2.3 times lower than in some other intermediate species including *Cleome paradoxa* (Marshall *et al.*, 2007), several *Flaveria* intermediates (McKown and Dengler, 2007) and *Salsola divaricata* (Voznesenskaya *et al.*, 2013). This may be explained by the *Portulaca* intermediates having much larger cells in succulent leaves.

In this study, most of the C_4_*Portulaca* species have higher vein density compared to three intermediate species. The species with Atriplicoid type of anatomy from both clades Umbraticola and Oleracea, have the highest vein density among all studied *Portulaca* types (see Fig. 3). Thus in the С_4_ Oleracea species, which have modified zig-zag Atriplicoid pattern of venation, the vein densities are ~1.8-fold higher than in the Cryptopetala intermediates (Fig. 3) and are very close to that published for flat-leaved C_4_*Cleome gynandra* (Marshall *et al.*, 2007); however, the densities in the Oleracea species is ~1.5 times lower than in C_4_*Flaveria* (McKown and Dengler, 2007). In C_4_ Pilosa species, which have peripheral vein distribution around terete or slightly flattened leaves with WS tissue in the middle, the vein densities were lowest among the C_4_ types analyzed. Compared to the intermediates, the vein densities on the adaxial side of the Pilosa species were ~1.3-fold higher than in the Cryptopetala intermediates, while the vein densities on the abaxial side of Pilosa species was similar to that in Cryptopetala intermediates. The Pilosa species may have a lower vein density than the Oleracea species, by having a higher volume of WS tissue and decrease in chlorenchyma tissue.

In contrast to the absence of clear correlation between vein density and the type of photosynthesis in succulent *Salsola* species (Voznesenskaya *et al.*, 2013), in most C_4_*Portulaca* lineages the vein density is higher than in the intermediates. However, among C_4_ lineages the vein density depends on the leaf anatomical type, with different patterns of minor veins distribution and/or forms of leaf succulence. In addition, in family Anacampserotaceae, which is closely related to the Portulacaceae, there is a C_3_ species *Anacampseros* (*Grahamia*) *coahuilensis* (with weak Crasulacean acid metabolism, Guralnik *et al.*, 2008) that has the same degree of succulence as *P. cryptopetala*, while its vein density is similar to that of C_4_ species in the Umbraticola and Pilosa (adaxial side) clades (5.0 ± 0.1 mm mm^−2^; Voznesenskaya and Koteyeva, unpublished data). This further supports the understanding that vein density in succulent lineages is more related to species-specific adaptations than to a progression change from C_3_ to C_4_ (Voznesenskaya *et al.*, 2013). Further analyses of leaf structure of different photosynthetic phenotypes in *Portulaca*, including vein density, volume of WS tissue and chlorenchyma tissue is needed to consider anatomical adaptations to arid environments.

### Evolutionary trends in types of photosynthesis

In the study of species in clade Cryptopetala, a variety of *P. cryptopetala* from Uruguay was analyzed (Table 1) in comparison to a variety from Argentina that was previously shown to be a C_3_-C_4_ intermediate species (Voznesenskaya *et al.*, 2010). While there are differences in plant morphology (the size of the plant, thickness and size of the stems and leaves, the size of flowers), the variety used in the current study also has a similar dorsoventral leaf anatomy. Analysis of the other species in the Cryptopetala clade, *P. hirsutissima* and *P. mucronata*, showed they also have C_3_-C_4_ anatomy with dorsoventral distribution of chlorenchyma. Microscopy studies show that BS cells in all three species have a substantial number of organelles with chloroplasts and enlarged mitochondria located mainly in a centripetal position. Measurements by gas exchange showed the values of Г in these three Cryptopetala species (20–32 μbar CO_2_) indicate functionally they are intermediates compared to the representative C_4_ species *P. oleracea*, which has a C_4_-type Γ value (2.6 μbar). Also, *P. cryptopetala* (variety from Argentina) has a Γ, which is intermediate to C_3_ (outgroup species *Sesuvium portulacastrum*) and C_4_*Portulaca* (Voznesenskaya *et al.*, 2010). C_4_ species typically have Г values of 0–5 μbar, C_3_ species ~60 μbar, and C_3_-C_4_ intermediates of 9–35 µbar depending on the species, e.g. Ku *et al.* (1991), Vogan *et al.* (2007).

The model for evolution of C_4_ plants developed from studies of species having reduced photorespiration consists of a stepwise progression of structural, biochemical and functional changes from C_3_ through stages of development of intermediates having a C_2_ cycle, followed by intermediates having increased acquisition of the C_4_ cycle, to fully functional C_4_ photosynthesis (Sage *et al.*, 2012; Voznesenskaya *et al.*, 2013; Schulze *et al.*, 2016). In the C_2_ cycle photorespiratory glycine produced in M cells is shuttled to BS cells for decarboxylation by GDC where photorespired CO_2_ is concentrated, enhancing its capture by BS Rubisco. In all three Cryptopetala species, GDC is selectively localized in mitochondria of BS cells, which is a distinguishing biochemical feature of intermediate species, while in C_3_ species the labeling is distributed about equally between M and BS mitochondria (Rawsthorne *et al.*, 1988). Analyses by western blots of C_4_ pathway enzymes showed that PEPC, PPDK and NAD-ME were detectable in all three intermediate species in the Cryptopetala clade, but in much lower levels compared to the C_4_ species. Also, Voznesenskaya *et al.* (2010) found the levels of these C_4_ enzymes in *P. cryptopetala* (variety from Argentina) were low, similar to the C_3_ species *S. portulacastrum.* The results suggest the capacity for C_4_ cycle activity in intermediates in this clade is low, which is consistent with leaf biomass having C_3_-like carbon isotope values. Thus, when CO_2_ is limiting they may have an increase in their efficiency of photosynthesis primarily by refixing photorespired CO_2_ in BS cells by the C_2_ cycle, with restricted contribution by a C_4_ cycle (Edwards and Ku, 1987; Ku *et al.*, 1991; Rawsthorne and Bauwe, 1998). Whether there is any C_4_ cycle activity in these species could be more directly analysed by the method of Alonso-Cantabrana and von Caemmerer (2016) via online measurements of photosynthesis and carbon isotope discrimination. The lack of identification of C_3_ and other species that could represent the proposed progression in C_4_ evolution in the genus *Portulaca* could suggest they have become extinct or have not yet been discovered.

All the *Portulaca* species in the study from the Pilosa clade were shown to have Kranz-type Pilosoid anatomy. One of them, *P. biloba*, has flattened subterete leaves while *P.* cf. *gilliesii, P. elatior, P. halimoides* and *P. smallii* have cylindrical terete leaves. In these species, the lateral VB with Kranz anatomy are distributed around the leaf periphery; while the main vein, which lacks a chlorenchyma sheath, is enclosed by WS tissue located in the central part of the leaf. That type of structure was classified as having multiple simple Kranz units (with many separate VB surrounded by two layers of Kranz tissue) according to classification by Peter and Katinas (2003). A similar leaf anatomy was shown for other representatives of this clade (Voznesenskaya *et al.*, 2010; Ocampo *et al.*, 2013). Some species in this clade were previously shown to have NADP-ME type of C_4_ biochemistry (Voznesenskaya *et al.*, 2010); in the present study additional species were shown to be NADP-ME type with one exception. *Portulaca elatior,* with Pilosoid leaf anatomy, was shown to have NAD-ME type of biochemistry, and it represents the earliest-divergent lineage of the Pilosa clade. This was surprising, since NAD-ME subtype was previously associated only with the Oleracea clade while all other C_4_*Portulaca* were considered NADP-ME subtype (Voznesenskaya *et al.*, 2010). The results suggest NAD-ME type of C_4_ biochemistry evolved twice within *Portulaca*, once in the Oleracea clade and once in *P. elatior* (Pilosa clade). It suggests evolution of different forms of C_4_ is more complex than previously thought. This discovery is consistent with the hypothesis that all photosynthetic modifications within *Portulaca*, including C_3_-C_4_ intermediates, were probably derived from C_4_ NADP-ME type based on phylogenetic analyses (Fig. 8 and Ocampo *et al.*, 2013). Previously it was shown the dominant form of photosynthesis in *Portulaca* is C_4_, which was possibly lost in species in the Cryptopetala clade. Similar cases, where intermediates are nested within C_4_ species, were shown for some clades in Aizoaceae (Bohley *et al.*, 2015) and Salsoleae (Schüßler *et al*., in press); which might be other cases where reversions from C_4_ to intermediates or C_3_ occurred. This is in contrast to studies in some families, which support evolution from C_3_-C_4_ intermediates to C_4_ photosynthesis (see *Leaf anatomy* in evolutionary trends section, this paper, and Sage *et al.*, 2012, 2014). Other studies suggest that a C_3_-C_4_ ancestor may have given rise to different C_4_ biochemical forms independently in separate lineages (e.g. Christin *et al.*, 2011). This may have been in response to specific environmental conditions, although the adaptive advantages of the different C_4_ variations are not well known. Careful large-scale analyses of the distribution of C_3_-C_4_ species and closely related C_3_ and C_4_ lineages showed it is impossible to define a universal ecological C_3_-C_4_ niche, nevertheless between different ecological factors temperature is one of the most important driving forces (Lundgren and Christin, in press). Likewise, the diversity within *Portulaca* could be facilitated by development of different photosynthesis strategies in separate lineages from unknown C_3_-C_4_ intermediate ancestors; although there is no evidence to support this. Gas exchange analyses indicate the C_3_-C_4_ intermediates (which have the CO_2_ concentrating C_2_ cycle) would be less capable of maintaining their photosynthesis under CO_2_ limited conditions than C_4_ species, which have the CO_2_ concentrating C_4_ cycle. Both intermediates and different Kranz forms of C_4_*Portulaca* have been found to grow together in some locations (Ocampo *et al.*, 2013). Further studies may help to clarify photosynthetic diversification in the different lineages of *Portulaca*, including the potential adaptive advantage of each phenotype based on habitat and seasonal growth cycles.

## Supplementary data

Supplementary data are available at *JXB* online.

S1. Electron microscopy of *in situ* immunolocalization of glycine decarboxylase (GDC) in M (panels A, C, E, G, I) and BS (panels B, D, F, H, J) cells of *Portulaca* species having different types of photosynthesis, and the C_3_ outgroup *Sesuvium portulacastrum*. Gold particles in M and BS cells in C_3_*S. portulacastrum* (A, B), C_3_–C_4_*P. hirsutissima* (C, D), C_3_–C_4_*P. mucronata* (E, F), C_3_–C_4_*P. cryptopetala* variety from Uruguay (G, H) and C_4_*P. oleracea* (I, J). Scale bars: 0.2 μm for C, D; 0.5 μm for A, B, E–H. c, chloroplast; m, mitochondria. Also, see Voznesenskaya *et al.*, (2010), which showed selective localization of GDC in mitochondria in BS cells of *P. cryptopetala* variety from Argentina, whereas the C_3_ outgroup species *S. portulacastrum* had equivalent labeling in M and BS mitochondria.

## Supplementary Material

Supplementary_Figure_S1

## Abbreviations:

BS: bundle sheath
δ^13^C value: a measure of the carbon composition
Γ: CO_2_ compensation point
GDC: glycine decarboxylase
M: mesophyll
NAD-ME: NAD-malic enzyme
NADP-ME: NADP-malic enzyme
PEPC: phosphoenolpyruvate carboxylase
PPDK: pyruvate,Pi dikinase
PPFD: photosynthetic photon flux density
Rubisco: ribulose bisphosphate carboxylase oxygenase
VB: vascular bundle(s)
WS: water storage tissue.
