# Supplementary material for: Unique photosynthetic phenotypes in Portulaca (Portulacaceae): C3-C4 intermediates and NAD-ME C4 species with Pilosoid-type Kranz anatomy
Source: J Exp Bot. 2016 Dec 15;68(2):225–39. doi: 10.1093/jxb/erw393 (PMC5853368; doi:10.1093/jxb/erw393)
Supplement: Supplementary_Figure_S1 [file erw393_suppl_supplementary_figure_s1.pdf]

**Supplementary data**

**Identification and characterization of unique photosynthetic phenotypes in *Portulaca* (Portulacaceae): $C_3$-$C_4$ intermediates and NAD-ME $C_4$ species with Pilosoid type Kranz anatomy.**

**Elena V. Voznesenskaya, Nuria K. Koteyeva, Gerald E. Edwards, and Gilberto Ocampo**

**Fig. S1.** Electron microscopy of *in situ* immunolocalization of glycine decarboxylase (GDC) in M (A, C, E, G, I) and BS (B, D, F, H, J) cells of *Portulaca* species having different types of photosynthesis, and the $C_3$ outgroup *Sesuvium portulacastrum*. Gold particles in M and BS cells in $C_3$ *S. portulacastrum* (A, B), $C_3$–$C_4$ *P. hirsutissima* (C, D), $C_3$–$C_4$ *P. mucronata* (E, F), $C_3$–$C_4$ *P. cryptopetala* variety from Uruguay (G, H) and $C_4$ *P. oleracea* (I, J). Scale bars = 0.2 µm for C, D; 0.5 µm for A, B, E-H. c, chloroplast; m, mitochondria. Also, see Voznesenskaya *et al.*, (2010) which showed selective localization of GDC in mitochondria in BS cells of *P. cryptopetala* variety from Argentina, whereas the $C_3$ outgroup species *S. portulacastrum* had equivalent labeling in M and BS mitochondria.

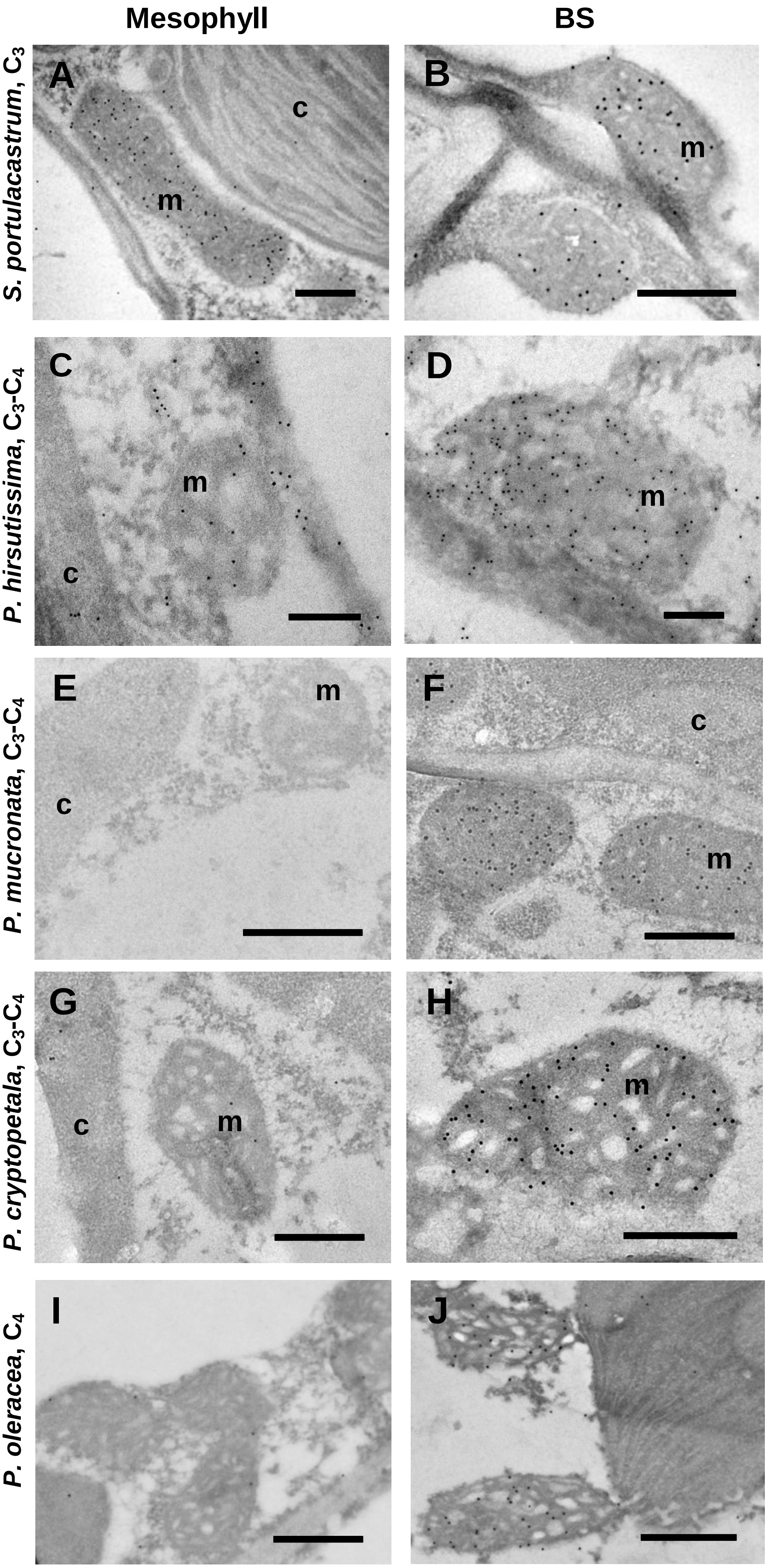

Mesophyll
BS
S. portulacastrum, C3
P. hirsutissima, C3-C4
P. mucronata, C3-C4
P. cryptopetala, C3-C4
P. oleracea, C4
A
B
C
D
E
F
G
H
I
J
m
c
